# Commentary on the impact of early exposure and mentorship on the neurosurgery career aspirations of medical students in LMICs

**DOI:** 10.1097/MS9.0000000000002806

**Published:** 2025-01-09

**Authors:** Ruth Wanjiku Mwangi, Kelvin Samuria Sokoto, Bartay Matui

**Affiliations:** Moi University School of Medicine, Eldoret, Kenya

Neurosurgery is a lifesaving intervention for people with serious brain and spinal disorders; however, access to neurosurgical care in low- and middle-income countries (LMICs) is limited due to the low number of neurosurgeons. This article gives an in-depth analysis of how early exposure to neurosurgery and mentorship play a role in influencing medical students’ career paths in LMICs. One of the strengths of this paper is the exploration of an important topic relevant to the development of neurosurgery in LMIC and the availability of adequate studies for a standard literature review. However, the paper has limitations such as financial constraints to access databases and excluding non-English articles^[1]^.

The article does a good job of drawing attention to the challenges faced by the neurosurgery personnel in LMICs. These include a scarcity of trained professionals, brain drain, and inequalities in training opportunities^[2,3]^. Furthermore, poor remuneration and inadequate facilities hamper the retention of skilled neurosurgical personnel^[4]^. It also adds that infrastructural development, providing more training opportunities and effecting competitive remuneration are vital in bolstering the neurosurgery workforce in LMICs.

The article primarily focuses on how early neurosurgical exposure bridges the neurosurgical knowledge gap by providing the technical skills required in clinical setups through global outreach programs and collaborations between high-income countries and LMIC^[5]^. Mentorship offers insightful critiques and guidance; hence, mentees have the necessary skills to navigate their careers. The authors suggest strengthening neurosurgery exposure in medical education through initiatives such as electives and incorporating mentorship into career development strategies to equip future neurosurgeons^[6]^.

Despite the advantages that result from mentorship and early neurosurgical exposure, the paper also highlights drawbacks such as the high cost of the program, duration of the program, fear of work-life balance, cultural influences, and personality traits that may influence motivation for pursuing neurosurgery^[7]^.

The analysis of this article would applaud its comprehensive investigations of the challenges and recommendations given to create a long-term neurological workforce in LMIC. However, it mostly focuses on the benefits of exposure and mentorship from an academic and professional perspective. Understanding the sociocultural and personal challenges that student’s face may give a better perception of difficulties in this field.
